# Role of CBFs as Integrators of Chloroplast Redox, Phytochrome and Plant Hormone Signaling during Cold Acclimation

**DOI:** 10.3390/ijms140612729

**Published:** 2013-06-18

**Authors:** Leonid V. Kurepin, Keshav P. Dahal, Leonid V. Savitch, Jas Singh, Rainer Bode, Alexander G. Ivanov, Vaughan Hurry, Norman P. A. Hüner

**Affiliations:** 1 Department of Biology and the Biotron Center for Experimental Climate Change Research, Western University, London, ON N6A 5B7, Canada; E-Mails: rbode3@uwo.ca (R.B.); aivanov@uwo.ca (A.G.I.); 2 Department of Plant Physiology, Umeå Plant Science Centre, Umeå University, Umeå 901 87, Sweden; E-Mail: vaughan.hurry@umu.se; 3 Department of Biological Sciences, University of Toronto Scarborough, Toronto, ON M1C 1A4, Canada; E-Mail: kdahal@utsc.utoronto.ca; 4 Eastern Cereal and Oilseed Research Centre, Agriculture and Agri-Food Canada, 960 Carling Avenue, Ottawa, ON K1A 0C6, Canada; E-Mails: leonid.savitch@agr.gc.ca (L.V.S.); jas.singh@agr.gc.ca (J.S.)

**Keywords:** CBF, cold acclimation, photosynthesis, redox imbalance, gibberellins, abscisic acid, phytochromes

## Abstract

Cold acclimation of winter cereals and other winter hardy species is a prerequisite to increase subsequent freezing tolerance. Low temperatures upregulate the expression of C-repeat/dehydration-responsive element binding transcription factors (CBF/DREB1) which in turn induce the expression of *COLD-REGULATED* (*COR*) genes. We summarize evidence which indicates that the integration of these interactions is responsible for the dwarf phenotype and enhanced photosynthetic performance associated with cold-acclimated and *CBF*-overexpressing plants. Plants overexpressing *CBFs* but grown at warm temperatures mimic the cold-tolerant, dwarf, compact phenotype; increased photosynthetic performance; and biomass accumulation typically associated with cold-acclimated plants. In this review, we propose a model whereby the cold acclimation signal is perceived by plants through an integration of low temperature and changes in light intensity, as well as changes in light quality. Such integration leads to the activation of the *CBF*-regulon and subsequent upregulation of *COR* gene and *GA 2-oxidase* (*GA2ox*) expression which results in a dwarf phenotype coupled with increased freezing tolerance and enhanced photosynthetic performance. We conclude that, due to their photoautotrophic nature, plants do not rely on a single low temperature sensor, but integrate changes in light intensity, light quality, and membrane viscosity in order to establish the cold-acclimated state. *CBFs* appear to act as master regulators of these interconnecting sensing/signaling pathways.

## 1. Introduction

Acclimation of cold-tolerant plant species to low, non-freezing temperatures increases the subsequent tolerance to freezing stress [1]. The biological variation exhibited by plant species with respect to their ability to cold acclimate and to develop freezing tolerance is genetically determined. Since plants are photoautotrophic, the ultimate energy source for the cold acclimation process is derived from the sunlight absorbed and transformed through the process of photosynthesis [2–5]. Cold or chilling stress refers to the exposure of plants to an abrupt shift from control, non-acclimated temperatures (20 to 25 °C) to low, non-freezing temperatures (0 to 5 °C) for a period of several hours to several days. Such a temperature shift induces a disruption of the plant’s overall homeostatic state. In contrast, cold acclimation is a long-term process occurring over several weeks to months where plant growth and development at a low, non-freezing temperature induces a new homeostatic state which typically is associated with a change in phenotype [3,4,6,7]. This new homeostatic state represents the cold-acclimated state. On the other hand, plant freezing tolerance is usually measured as LT50, the freezing temperature at which 50% of a plant population dies due to a short freeze stress event. This review is focused on the processes involved in establishing the new cold-acclimated state in cold-tolerant plants and photosynthetic microorganisms.

The process of cold acclimation is regulated by C-repeat/dehydration-responsive element binding transcription factors (CBF/DREB1) [8–15]. CBF/DREB1 transcription factors bind to the C-repeat/dehydration-responsive DNA regulatory elements in the promoters of *COLD-REGULATED* (*COR*) genes [14,16–18]. In *Arabidopsis thaliana* (L.) Heynh, cold acclimation or cold stress activate expression of three *CBF* genes (*CBF1/DREB1b*, *CBF2/DREB1c*, and *CBF3/DREB1a*) which, in addition to other genes, induce the expression of several *COR* genes [14,16,17,19–23]. Studies with the *A. thaliana CBF2* null mutant in a Colombia (Col) background have shown that the freezing tolerance and the expression of *CBF1* and *CBF3* were increased by the absence of *CBF2* [24], thus suggesting that *CBF2* negatively regulates the expression of *CBF1* and *CBF3* [25]. However, another study which used *A. thaliana* plants from the Wassilewskija-2 (WS-2) background reported that all three CBF genes were positively involved in freezing tolerance and activation of *COR* genes [17].

The expression of *CBF3* is positively regulated by the constitutively expressed ICE1 (inducer of *CBF* expression 1) gene, the product of which binds to multiple regulatory elements present in the *CBF3* promoter and stimulates its transcription [8,12,14,26]. The action of ICE1, in turn, is tightly regulated by SIZ1, a SUMO (small ubiquitin-related modifier) E3 ligase, and HOS1 (high expression of osmotically responsive protein 1) which is a RING finger E3 ligase [12,14,27–29]. In non-acclimated *A. thaliana* plants, the HOS1 protein, which is located in the cytoplasm, causes ubiquitination and degradation of ICE1 [27,28]. However, when *A. thaliana* plants are exposed to low temperature conditions, the HOS1 protein relocates to the nucleus [28] and the SIZ1 protein sumoylates ICE1 protein which, in turn, activates At*CBF3* expression [29].

Overexpression of *CBF* genes in *A. thaliana*, canola (*Brassica napus* L.), tomato (*Solanum lycopersicum* L.) and poplar (*Populus balsamifera* subsp. *trichocarpa*) increased their freezing tolerance [16,30–32]. Interestingly, the increased freezing tolerance of *CBF*-overexpressing plants is associated with a dwarf phenotype and delayed flowering [16,30,31,33,34]. Similarly, cold acclimation of WT plants results in a dwarf phenotype [30,31,33,34]. Thus, the CBF regulon-induced freezing tolerance is associated with phenotypic changes that are analogous to growth and development events that are controlled by plant hormones and photoreceptors such as phytochromes [35]. In *A. thaliana*, there are five phytochrome genes (*PHYA*, *PHYB*, *PHYC*, *PHYD* and *PHYE*) [36]. Three of them, *PHYB*, *PHYD* and *PHYE* are involved in regulation of plant growth by light quality (red to far-red ratio) [37–40]. The *phyB*-deficient *A. thaliana* displays increased elongation, decreased leaf expansion, increased apical dominance, and early flowering [37]. *PhyA* is involved in light quality-regulated seed germination [41] and phyC is suspected to be involved in the red light inhibition of hypocotyl elongation [40,42]. Interestingly, *A. thaliana* WS ecotype was found to be a natural *phyD* deletion mutant [43], and Col and Landsberg erecta (Ler) ecotypes are known to exhibit divergent growth and development in response to light quality [44,45].

Plant growth and development is regulated by multiple plant hormones: gibberellins (GAs), auxins, cytokinins (CKs), brassinosteroids (BRs), abscisic acid (ABA), ethylene and, potentially, salicylic acid and jasmonic acid [46]. While the individual roles of plant hormones have long been established, they often overlap, because most, if not all, plant growth and development processes are regulated by several plant hormones via positive and negative interactions [47]. For example, auxins interact with GAs by upregulating the *GA20ox* and *GA3ox* expression, while downregulating *GA2ox* expression [48]. Cold acclimation of most plant species is associated with enhanced freezing tolerance and the phenotype of cold-acclimated plants is often characterized by dwarfism or, at least, a compact, rosette growth habit [14]. The classic example of the GA response is the dramatic stem elongation induced in short-day, rosette cabbage plants by the application of exogenous GA [49]. Thus, a dwarf or compact growth habit is a response to a decrease in endogenous GA levels [49]. Plants overexpressing *CBF* genes exhibit a dwarf phenotype regardless of the temperature treatment in *A. thaliana* [33], canola [31] and tomato [30]. Thus, the process of cold acclimation must involve changes in hormonal homeostasis. First, we dissect the involvement of plant hormones in cold acclimation, cold stress and freezing tolerance, and evaluate their interaction with the *CBF* regulon.

Second, we reported recently that overexpression of CBFs in *Brassica napus* cv. Westar not only induces a dwarf phenotype with enhanced freezing tolerance without prior exposure to low temperature, but concomitantly, also induces increased photosynthetic performance and water use efficiency combined with enhanced photosynthetic energy conversion efficiency and biomass accumulation [31,50,51]. In fact, unlike most dwarf plants, cold-acclimated plants exhibit a total plant biomass that is equal to or greater to control plants that exhibited the elongated phenotype [6,52,53]. Thus, we attempt to integrate the sensing of low temperatures via photosynthetic redox imbalance quantified as excitation pressure with the role(s) of *CBFs*, plant growth hormones and phytochromes in the governance not only of cold acclimation, phenotypic plasticity and freezing tolerance, but also photosynthetic performance and energy conversion efficiency.

## 2. CBF–Hormone Interactions, Cold Acclimation and Dwarf Phenotype

### 2.1. Gibberellins

**Figure 1:**
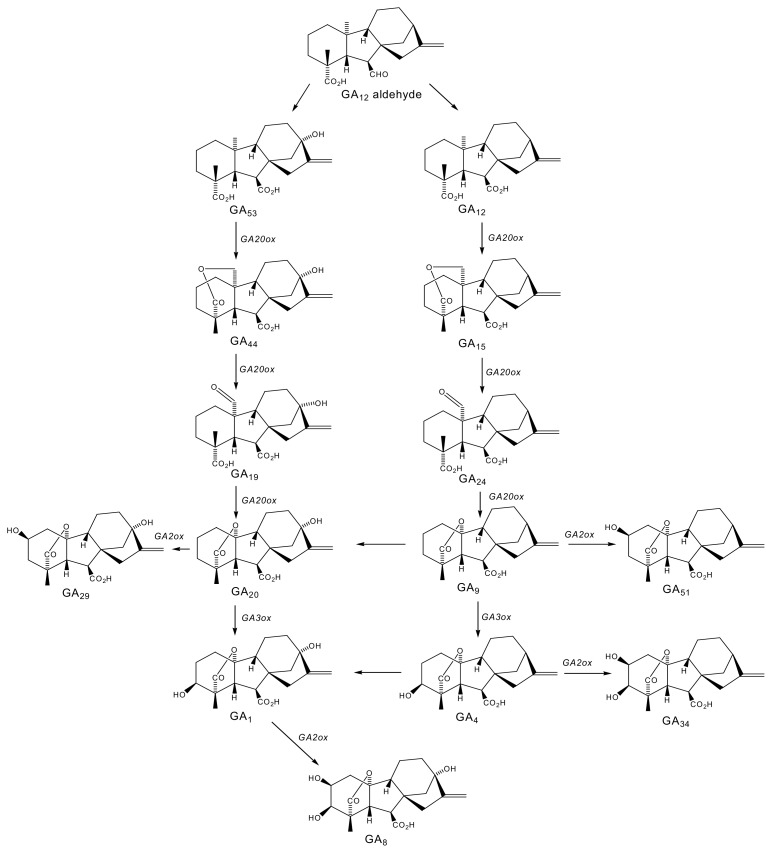
Pathways of gibberellin biosynthesis: two putative pathways of gibberellin biosynthesis, the early 13-hydroxylation and early non-hydroxylation pathways that are likely utilized in vegetative tissues of many higher plants [48,54,70]. The GA_9_→GA_20_ and GA_4_→GA_1_ conversions are based on a limited number of examples in the literature, and thus may not be commonplace. Compliments of Ruichuan Zhang and Richard Pharis (University of Calgary, Calgary, AB, Canada).

The application of exogenous growth-active GAs (such as GA_1_, GA_3_ or GA_4_) enhances shoot growth and mutants that are deficient in endogenous GAs, or have impaired GA signaling, exhibit a dwarf phenotype. Thus, GAs are promoters of stem elongation [48,54,55]. The biosynthetic pathway of GAs involves genes encoding for each of GA20-oxidase (GA20ox), GA3-oxidase (GA3ox) and GA2-oxidase (GA2ox) enzymes that control oxidation steps involving C-20 and each of the C-3β and C-2β-hydroxylations (Figure 1) [48]. *A. thaliana* plants overexpressing *GA20ox* or *GA3ox* produce higher levels of growth-active GAs and exhibit a large, extended phenotype [54]. In contrast, plants overexpressing *GA2ox* which is responsible for catabolism of growth-active GA_1_ and GA_4_ to their catabolite forms, GA_8_ and GA_34_ respectively, and catabolism of precursors to growth-active GAs, GA_20_ and GA_9_ to their catabolite forms, GA_29_ and GA_51_, respectively (Figure 1), exhibit a dwarf phenotype relative to wild-type (WT) plants [48,54,55]. It is well established that a change in temperature modifies endogenous GA levels [56–58], as well as the plant’s sensitivity to the application of exogenous growth-active GAs [59]. Endogenous GA levels decrease as temperature is lowered and this is associated with decreased shoot growth in carrot (*Daucus carota* L.) [60], wheat (*Triticum aestivum* L.) [61], *Campanula isophylla* [62], *Dendranthema grandiflorum* [63] and sunflower (*Helianthus annuus* L.) [58].

Surprisingly, initial transcriptome microarray studies of *A. thaliana* seedlings exposed to low (4 °C) temperature indicated that genes involved in GA biosynthesis were not cold responsive [64–66]. However, Achard *et al.* [33] did show that exposure of *A. thaliana* plants to a 4 °C cold stress caused an increase in the expression levels of *AtGA2ox3* and *AtGA2ox6*. Concomitantly, the cold-stressed plants exhibited lower endogenous levels of GA_1_ and GA_4_, and higher endogenous levels of GA_8_ and GA_34_[33]. It is important to note that the cold acclimation process is initiated only when the treatment temperature is lower than 10 °C. Exposure of *A. thaliana* seedlings initially grown at 22 °C to a lower temperature of 12 °C resulted in increased expression of the transcript level of *AtGA3ox1* and decreased transcription of *AtGA2ox2* [67]. Similarly, sunflower seedlings grown at 15 °C had higher endogenous GA_1_ levels and lower endogenous GA_8_ levels relative to seedlings grown at 20 °C [58].

Likewise, cold stress regulation of GA biosynthesis can differ as a function of a plant developmental stage. Exposure of imbibed *A. thaliana* seeds to a stratification temperature of 4 °C caused an increase in the expression of *AtGA3ox1* transcript levels and was associated with increased endogenous GA_1_ and GA_4_ levels [68]. This is expected since stratification results in “germination” of seeds which would otherwise have remained “dormant”. However, this is contrary to cold regulation of GA biosynthesis in seedlings [33]. A comparison of *CBF1* expression between maturing *A. thaliana* seeds and seedlings subjected to 4 °C cold treatment showed that, unlike seedlings, the maturing seeds did not increase their *CBF1* expression [69]. However, there was an upregulation of *AtGA2ox6* expression in maturing WT seeds subjected to cold treatment which was reduced in loss-of-function *CBF* lines [69]. Furthermore, no *CBF1* expression was detected in imbibed seeds prior to the same cold treatment as received by mature seeds and seedlings [69]. Following the initiation of cold treatment, the imbibed seeds did eventually show *CBF1* expression but with a lag in time and at significantly lower levels compared to that observed in seedlings. Concomitantly, the expression of *COR15b* in mature or imbibed seeds was not upregulated as it was in seedlings following a cold treatment [69].

The dwarf phenotype of *CBF*-overexpressing plants can be rescued by application of exogenous growth-active GAs, but not by application of other hormones which have been implied in shoot growth enhancement. Transgenic tomato plants overexpressing *A. thaliana CBF1* are cold-tolerant and dwarf, the latter can be rescued by exogenous application of GA_3_[30]. Interestingly, the *CBF1*-overexpressing tomato plants treated with GA_3_ and thus exhibiting a normal, elongated phenotype retained the same cold tolerance as non-GA_3_-treated *CBF1* plants [30]. However, it is likely that a reduction in the growth-active GA levels is necessary for cold or freezing tolerance since *A. thaliana* plants impaired in GA downstream signaling had reduced freezing tolerance. *A. thaliana* plants overexpressing *CBF1* grown to the same developmental stage at 22 °C had increased *AtGA2ox3* and *AtGA2ox6* expression, as well as higher GA_8_ and GA_34_, and lower GA_1_ and GA_4_ levels [33]. Application of exogenous GA_3_ to *CBF1* overexpressing *A. thaliana* plants rescued the dwarf phenotype [33]. Overexpression of cotton (*Gossypium hirsutum*)-derived *GhDREB1* in *A. thaliana* delayed vegetative growth and flowering [34]. The initial, but not subsequent flowers, exhibited symptoms of male sterility such as shorter filaments and undehisced anthers relative to the WT type line [34]. Exposure of plants from this overexpressing line to low temperature resulted in higher tolerance to freezing stress than the WT. The dwarf phenotype of the *A. thaliana* line overexpressing *GhDREB1* was rescued by exogenous application of GA_3_, but not by applications of growth-active auxin, BR or CK [34].

Phylogenetic studies in *A. thaliana* have shown that genes encoding the *CBF* family of transcription factors are closely related to *dwarf and delayed-flowering1* (*DDF1*) gene [71]. Expression of *RD29A/COR78* was also upregulated 9-fold in *ddf1* plants, but not the expression of *CBF3* genes [71]. Furthermore, both *CBF* and *DDF1*-overexpressing plants exhibit dwarf phenotypes [71–73]. Mutant *ddf1* plants were reported to be GA-deficient and the dwarf phenotype could be rescued by application of exogenous GA_3_[71]. Furthermore, the endogenous GA_1_ and GA_4_ levels in *ddf1* plants were lower than in WT background lines due to the repression of *GA20ox* genes [71]. A latter study by the same group confirmed that *ddf1* plants have higher expression of *AtGA2ox7* relative to WT [74]. Similarly, *A. thaliana* plants transformed with grape *CBF1* (*VrCBF1*) exhibited increased expression of cold-regulated genes (*AtCOR15a*, *AtRD29A*, *AtCOR6.6* and *AtCOR47*) and *AtGA2ox7* which was associated with a dwarf phenotype and delayed flowering [75]. The latter was also associated with increased expression of *AtFLC* (*FLOWERING LOCUS C*), a floral repressor gene [75], confirming an earlier report of increased *AtFLC* expression and delayed flowering in *A. thaliana* plants overexpressing *GhDREB1* [34].

There appears to be an inverse relationship between endogenous GA levels and *CBF* expression: an increase in CBF expression reduced the accumulation of growth-active GAs [33,75]. Furthermore, the application of exogenous GA_3_ to cotton seedlings exposed to 0 °C for 24 h inhibited the increase in expression of *GhDREB1* observed for control seedlings [76]. Also, cold stress tolerance was strongly associated with increased expression of *CBF1* and lower endogenous GA_1_ and GA_4_ levels in four bamboo species that exhibited natural variation in low temperature sensitivity [77]. The current model of *CBF*-GA interaction proposes that overexpression of CBF, either via cold induction or by transgenic means, stimulates the accumulation of DELLA proteins [33]. These growth-repressing proteins are positioned downstream in the GA signaling pathway and are deactivated by accumulation of growth-active GAs which, in turn, leads to an elongated phenotype [78]. The accumulation of DELLA proteins is likely a consequence of a reduction in growth-active GA levels caused by increased expression of *GA2ox* genes. Thus, the evidence is consistent with the thesis that CBF controls the expression of G*A2ox* genes in cold-acclimated plants [33].

### 2.2. Abscisic Acid

Involvement of ABA in plant cold acclimation and freezing tolerance has been known for some time due, in part, to the observations that the application of exogenous ABA could substitute for exposure to cold stress [79–85] and that cold stress induces increases in endogenous ABA levels [77,80,86–88]. Furthermore, *A. thaliana* mutant lines deficient or impaired in ABA biosynthesis showed reduced response to cold acclimation [88–90]. Nevertheless, there are also conflicting reports where the application of ABA to a wide range of plant species did not induce cold hardiness or freezing tolerance [91–95]. Thus, while cold-acclimation or cold-stress events usually do increase endogenous ABA levels, the role of ABA in these events remained unclear. The involvement of ABA in activating a cascade of downstream signaling events in response to a cold treatment has been evaluated. In *A. thaliana* plants, both cold treatment and application of exogenous ABA increases the expression of several *COR* genes including *RAB18*, *RD29A* (also known as *COR78* or *LTI78*), *KIN2* [92,96,97], as well as *CBF1* [98]. Furthermore, in freezing-tolerant wild grapes (*Vitis riparia* Michx.) and freezing-sensitive cultivated grapes (*Vitis vinifera* L.) both cold (4 °C) and exogenous ABA treatments similarly activated the expression of *CBF1* gene [99]. In contrast, none of the *CBF* genes responded strongly to exogenous ABA treatment in barley (*Hordeum vulgare* L.) [100]. The current consensus is that the activation of *CBF* and *COR* gene expression can be both ABA-dependent and ABA-independent [101–103].

However, even in the scenario of ABA-independent cold-induced activation of *CBF* gene expression [99,101–103], low temperatures do increase endogenous ABA accumulation [77,81,86]. This increase in endogenous ABA levels in cold-acclimated or cold-stressed plants can play a role in the resultant dwarf phenotype as well as cause the dwarf phenotype of *CBF*-overexpressing plants grown at optimal growth temperatures [31,51] since elevated ABA levels are usually associated with a decrease in shoot growth [104–106]. Historically, GAs and ABA have been reported to act antagonistically in the regulation of shoot growth and both plant hormones can downregulate each other’s biosynthetic genes [107]. In *A. thaliana*, ABA inhibits the expression of *GA20ox* and *GA3ox* during seed germination, and thus, reduces the levels of growth-active endogenous GAs [108]. In sunflower hypocotyls subjected to a range of low temperatures, there was an antagonistic association between endogenous ABA and GA_20_ and GA_1_, but not GA_8_ levels which would imply an antagonistic association between ABA, and *GA20ox* and *GA3ox* genes [58]. Similarly, in four bamboo species, cold tolerance was strongly associated with increased expression of *CBF1* gene, higher endogenous ABA levels and lower endogenous GA_1_ and GA_4_ levels [77]. Thus, while the low temperature-mediated increase in *CBF* expression may occur independently of ABA, this plant hormone may still contribute to the reduced growth and the dwarf phenotype in *CBF*-overexpressing plants through its antagonistic interaction with CBF-dependent GA biosynthesis.

### 2.3. Cytokinins

Cytokinins (CKs) are involved mainly in the stimulation of cell division, lateral bud or shoot growth, leaf expansion and the prevention or deceleration of senescence [109]. Based on studies with CK-deficient plants it was postulated that CKs may act as negative regulators of abiotic stress signaling [110]. Abiotic stress can decrease CK content. A reduction in temperature stimulated the activity of cytokinin-oxidase and lowered endogenous CK levels in wheat [111]. *A. thaliana* plants with reduced CK biosynthesis due to the inactivation of the *IPT* gene were more tolerant to drought and salt stress than WT [112]. Also, these CK-deficient plants had reduced ABA levels and an increased sensitivity to exogenously applied ABA [112]. Thus, it is likely that CK homeostasis may play a role in plant responses to stress via changes in both ABA biosynthesis and the sensitivity of the plant to ABA [110].

The CK signal transduction pathway in *A. thaliana* involves histidine kinases (AHKs), which, upon activation, transfer the signal, via histidine phosphotransfer proteins (AHPs), to response regulators (ARRs), type-A and type-B [113,114]. Type-A response regulators inhibit type-B transcription factors through protein–protein interactions, whereas phosphorylation of type-B transcription factors regulates the expression of type-A ARRs and other genes [113,114]. *A. thaliana* plants overexpressing *GhDREB1* were shown to be less sensitive to the exogenous addition of the cytokinin, benzyladenine (BA) [34]. Furthermore, plants overexpressing *GhDREB1* had reduced expression of type-A and type-B ARR genes, whereas endogenous CK levels were not changed. This implies that *GhDREB1* exerts a negative regulation of downstream CK signaling without affecting CK biosynthesis [34].

*A. thaliana* plants of the *amp1* mutant line with increased endogenous CK levels [115] performed better than WT plants under continuous cold stress conditions by significantly increasing cell division [116]. The application of exogenous CK, zeatin, to WT plants mimicked the cold-induced *amp1* phenotype under continuous cold stress conditions. However, untreated and zeatin-treated WT as well as *amp1* plants exhibited similar levels of *CBF* and *COR* gene expression under continuous cold stress conditions [116]. Both cold stress and exogenous BA induced the expression of a subset of type-A *ARR* genes, whereas only cold stress, but not BA, induced the expression of *CBF1*, *RD29A*, and *RD29B* [117]. However, the cold-induced expression of type-A *ARR* was not associated with changes in endogenous CK levels. Furthermore, cold treatment of *35S:AtCKX2-2* plants with reduced CK levels did not result in changes of type-A *ARR* or *CBF* gene expression relative to wild-type plants [117]. Based on expression levels of type-A *ARR* and *CBF* in *ahk* single and double mutant lines, it was suggested that at least two CK-related *AHKs*, *AHK2* and *AHK3*, are the mediators of cold stress response which activates expression of a subset of type-A *ARR* genes, whereas *CBF* expression is not connected to *AHK* or *ARR* expression [117]. Also, overexpressing *ARR7* resulted in reduced freezing tolerance and insensitivity to applied ABA, whereas *arr7* plants exhibited increased tolerance to freezing stress and hypersensitivity to ABA. This was further linked to ABA and it was concluded that *AHK2*, *AHK3* and type-A *ARRs* inhibit ABA signaling, and thus, are negative regulators of the cold stress response in *Arabidopsis thaliana* [117]. In contrast, a more recent study reported that overexpression of *ARR7* in *Arabidopsis thaliana* exhibited higher freezing tolerance than WT [118]. It appears that further research is needed to confirm a definitive relationship between CK levels and *CBF* expression.

### 2.4. Ethylene

Ethylene plays an important role in plant responses to a range of biotic and abiotic stresses [119]. Plant response to stress often causes increased ethylene production which is likely causal for shoot growth inhibition [119–121]. Increased ethylene biosynthesis has been implied in the enhancement of chilling or freezing tolerance in cucumber (*Cucumis sativus* L.), sunflower, tobacco (*Nicotiana tabacum* L.), tomato and winter rye (*Secale cereal* L.) [58,122–125]. Exogenous application of ethylene’s immediate precursor, 1-aminocyclopropane-1-carboxylic acid (ACC), to tobacco plants promoted freezing tolerance, whereas application of the ethylene biosynthesis inhibitor, aminoethoxyvinylglycine (AVG) or the ethylene receptor antagonist, AgNO_3_, decreased freezing tolerance [125]. In contrast, ethylene production was decreased by cold treatment in winter wheat and dwarf beans (*Phaseolus vulgaris* L.) [126,127]. Furthermore, reduction in ethylene production was associated with increased freezing tolerance in both mung bean (*Vigna radiate* L.) and *A. thaliana* [118,128]. The *A. thaliana ethylene overproducer1* (*eto1*) mutant also exhibited reduced freezing tolerance relative to WT [118]. Furthermore, application of ACC to WT *A. thaliana* plants reduced freezing tolerance, whereas application of either AVG or AgNO_3_ increased freezing tolerance [118]. In line with the latter results, several ethylene insensitive mutant lines also exhibited enhanced freezing tolerance [118]. Therefore, the role of ethylene in cold acclimation and freezing tolerance appears to be complex and probably species dependent.

There is limited information on the potential interaction of ethylene with the *CBF* regulon, but this interaction may be negative. Plants overexpressing *ETHYLENE INSENSITIVE3* (*EIN3*), a transcription factor positively regulating ethylene signaling [129–131], exhibited reduced freezing tolerance and had decreased *CBF* expression whereas *ein3* plants exhibited increased freezing tolerance and increased expression of *CBF* relative to WT [118]. Furthermore, overexpression of *CBF* in *A. thaliana* also suppresses the responsiveness of leaves to ethylene, therefore delaying senescence and chlorophyll degradation [132]. Also, ethylene was suggested to be a negative regulator of CK signaling. Shi *et al.* reported that ethylene inhibited the expression of *CBF* and type-A *ARR* genes involved in CK signaling which caused reduced freezing tolerance [118].

### 2.5. Brassinosteroids

Brassinosteroids (BR) are mainly recognized for their involvement in stem etiolation [133], although applied growth-active BR, brassinolide (BL), can also promote stem elongation in light-grown plants [134–136]. There are several examples in the literature of exogenous BRs alleviating the effects of heat stress [135,137,138], likely via upregulation of ABA biosynthesis [139]. However, the involvement of BRs in the alleviation of cold stress is not well understood. Pretreatment with growth-active BRs did alleviate the effect of cold stress on chlorophyll content in corn (*Zea mays* L.) and increased *A. thaliana* tolerance to cold treatment [140–142]. Furthermore, the *brassinosteroid-insensitive 1* (*bri1*) mutant with deficiency in BR signaling [143,144] was more tolerant, whereas the *BRI1*-overexpressing line was less tolerant to cold treatment compared with WT [142]. Both *bri1* and *BRI1* lines had higher basal expression levels of *CBF* genes under control temperature relative to WT [142]. Also, application of BR to non-stressed plants promoted the expression of *COR15A* [145]. Although this may suggest an involvement of BRs in CBF regulation, the cold-treated WT type, *bri1* and *BRI1* lines all showed similar increases in expression of both *CBF* and *COR* genes [142]. In contrast, *A. thaliana* seedlings overexpressing the BR biosynthetic gene *AtDWF4* [146] were more tolerant to cold stress treatment and had higher expression of the *COR15A* gene [145]. Thus, a role of BRs in the regulation of CBFs and cold tolerance appears to be equivocal. Further research is required to elucidate the role of BRs in CBF regulation of cold tolerance.

### 2.6. Salicylic Acid

Salicylic acid (SA) has been mainly associated with the activation of plant defense mechanisms against pathogen attack [147]. However, application of exogenous SA can affect the physiology, metabolism and reproductive development of some higher plants [148]. At higher exogenous concentrations, SA often inhibits growth [149], but at low, near-physiological concentrations it may promote biomass accumulation [150]. Pretreatment of plants with exogenous SA effectively alleviated some of the cold stress-induced effects in several species such as banana (*Musa acuminate* L.), corn, cucumber, peach (*Prunus persica* [L.] Batch.), potato (*Solanum tuberosum* L.), rice (*Oryza sativa* L.) and tomato plants [151–160]. Cold treatment increased endogenous SA accumulation in *A. thaliana* WT, but mutant lines with reduced SA biosynthesis (*NahG* and *eds5*) responded to cold treatment with increased growth relative to WT [116,161–163]. In contrast, the SA overproducing line, *cpr1*, was less tolerant to cold stress and exhibited acceleration in the development of a dwarf phenotype [163]. However, the expression of *CBF* genes in cold-treated WT and *NahG* mutant lines was similar [116]. A study with the SA overproducing lines, *siz1* and *acd6* which exhibit a dwarf phenotype, confirmed the decrease in tolerance to cold stress associated with higher endogenous SA accumulation and linked it to a decrease in *CBF* gene expression [164].

## 3. Light, Cold Acclimation and Freezing Tolerance

The light-dependence of plant cold acclimation and freezing tolerance is not new [6,165]. However, confusion persists in the literature with respect to the role of *light quality*, sensed through plant photoreceptors such as phytochrome and cryptochrome, *versus* the role of *light intensity*, sensed through redox imbalances associated with photosynthetic electron transport. The former initiates and governs processes involved in plant photomorphogenesis while the latter process results in alterations in the structure and function of the chloroplast photosynthetic apparatus through the process of photoacclimation [2,166–170]. When uncoupled, both light quality and light intensity signals were shown to differently regulate plant hormone biosynthesis and thus plant phenotype [171,172]. Walters *et al.* [173] reported that photomorphogenic mutants of *A. thaliana* exhibited photoacclimation and concluded that photoreceptors do not play a major role in photoacclimation. However, a *det1* signal transduction mutant did exhibit a defect in photoacclimation from which they concluded that there must be cross-talk between photoreceptor-regulated responses and regulatory components involved in photoacclimation in *A. thaliana* [173].

### 3.1. Light Quality, Photoreceptors and Cold Acclimation

**Figure 2:**
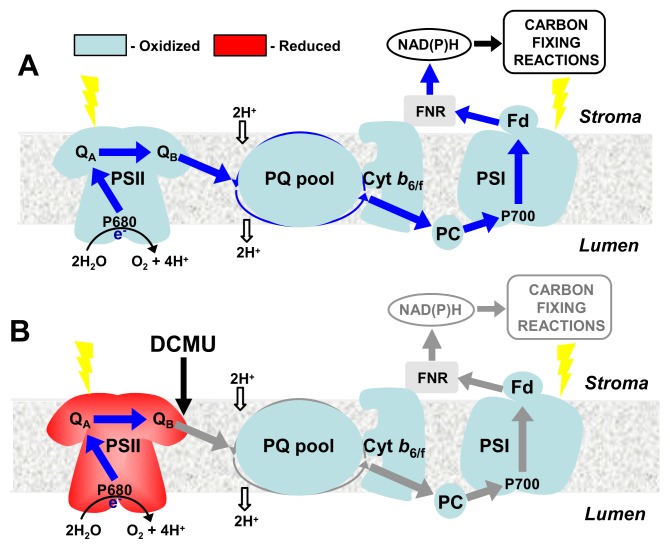

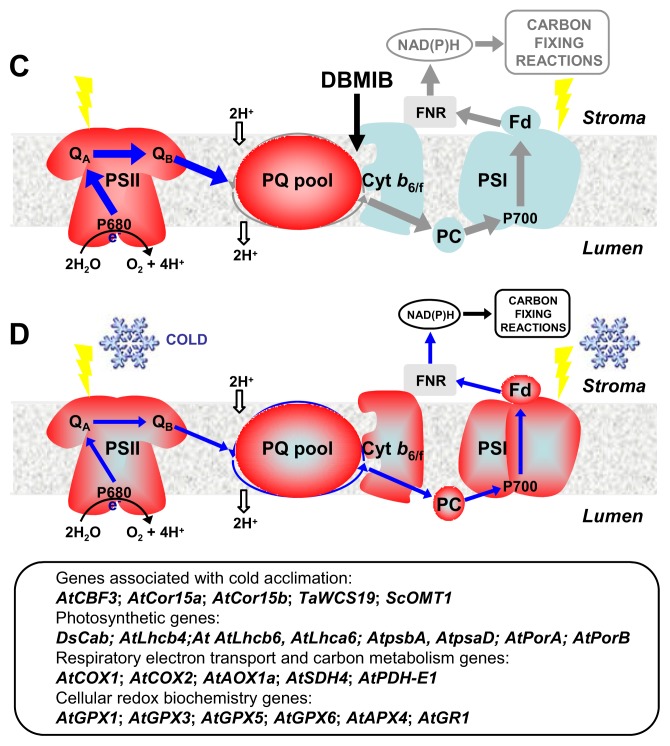
Simplified overview of possible effects of specific inhibitors and cold stress on the redox state of the photosynthetic electron transport chain components. (**A**) During growth and development of plants under optimal temperature conditions, the PQ pool and all components of the photosynthetic electron transport chain remain preferentially oxidized (light blue) because the rate of consumption of photosynthetic electrons through metabolic sinks (carbon fixing reactions) keeps pace with the rate at which PSII undergoes charge separation to reduce the PQ pool. Under these conditions, the linear photosynthetic electron flow (dark blue arrows) from PSII (water splitting) to PSI (NADP^+^ generation) dominates and is fully operational; (**B**) Adding DCMU [3-(3,4-dichlorophenyl)-1,1-dimethylurea], a selective inhibitor at the Q_B_ binding site of PSII, to chloroplast membranes or intact leaves, blocks the linear electron transport rendering photosystem II (PSII) components more reduced (red), while all components downstream of PSII remain oxidized due electron consumption by photo-oxidized PSI; (**C**) The specific inhibitor of Cytb*_6/f_* complex (Q cycle) DBMIB (2,5-dibromo-3-methyl-6-isopropyl-p-benzoquinone) causes a reduction of PSII complex as well as the PQ pool, whereas the components downstream of PQ remain oxidized; (**D**) Exposure of plants to cold stress results in lower demand for electrons required for carbon fixing reactions. Cold stress imposes thermodynamic limitations in the rates of consumption of photosynthetically-generated electrons by the carbon fixation reactions on the acceptor side of PSI which increases the reduction state of PQ pool and all components of the photosynthetic electron transport chain. Such a reduction state is quantified *in vivo* as excitation pressure using chlorophyll fluorescence induction. Excitation pressure reflects the relative redox state of Q_A_, the first stable quinone electron acceptor in the PSII reaction centre. Under cold stress conditions (Figure 2D), the linear photosynthetic electron flow between PSII and PSI is partially restricted relative to controls (Figure 2A).

Pogson and co-workers [174,175] differentiate sensing/signaling associated with changes in light quality through photoreceptors involved in photomorphogenesis as “biogenic signals” *versus* sensing/signaling associated with changes in light intensity through photosynthetic electron transport in mature chloroplasts as “operational signals”. For example, the former are involved in chloroplast biogenesis and govern the proper biosynthesis and assembly of thylakoid membranes and their constituent membrane protein complexes whereas the latter are required to ensure energy balance and cellular homeostasis in an environment where light intensity, temperature, water and nutrient availability constantly fluctuate [4,5,176]. Kim *et al.* reported that phytochrome regulates low temperature-induced gene expression of *COR15a* through CBFs in *A. thaliana* [177]. In an attempt to eliminate the possible role of light-mediated gene expression through photosynthesis, these authors reported that inhibition of photosystem II (PSII) photochemistry by DCMU [3-(3,4-dichlorophenyl)-1,1-dimethyl urea] did not affect *CBF* gene expression. On this basis, they concluded that changes in photosynthetic redox potential did not contribute to the light regulation of *CBF* and *COR* gene expression during cold acclimation of *A. thaliana* [177]. However, the experimental design employed by Kim *et al.* [177] is seriously flawed for the following reasons. First, the reduction state of the PQ (plastoquinone) pool of the photosynthetic electron transport chain is considered an important sensor which governs nuclear gene expression through retrograde signaling [174,178–182]. In the presence of DCMU, although electron transfer from PSII to the PQ pool is inhibited, the PQ pool is oxidized as a consequence of electron transfer from the PQ pool to PSI via the cytochrome b_6_/f complex and plastocyanin (Figure 2B) [4,5]. To assess the effects of a reduced PQ pool, one must measure the effects of the electron transport inhibitor, DBMIB (2,5-dibromo-3-methyl-6-isopropylbenzoquinone) which is known to block photosynthetic electron transport at the cytochrome b_6_/f complex [4,5]. Thus, in the light and in the presence of DBMIB, the PQ pool remains reduced because PSII transfers electrons into the PQ pool, but electrons cannot exit the PQ pool because electron transfer through the cytochrome b_6_/f complex to PSI is blocked (Figure 2C). Thus, one must modulate the redox state of the PQ pool with both DCMU and DBMIB separately. Second, in addition to the PQ pool, the acceptor-side of PSI is also an important generator of redox signals that emanate from photosynthetic electron transport [5,183,184]. Since Kim *et al.* [177] failed to test the effects of DBMIB on *CBF* expression, the conclusions of their photosynthetic electron transport inhibition studies remain equivocal. In Figure 2D, we present a few specific examples of photosynthetic and non-photosynthetic genes shown to be regulated by changes in the redox state of the photosynthetic electron transport chain in wheat (*Triticum aestivum*, *Ta*), rye (*Secale cereals*, *Sc*), *Dunaliella salina* (*Ds*), and *Arabidopsis thaliana* (*At*). These include photosynthetic (*DsCab; AtLhcb4;At AtLhcb6*, *AtLhca6; AtpsbA*, *AtpsaD; AtPorA; AtPorB*) as well as non-photosynthetic genes previously associated with cold acclimation (*AtCBF3; AtCor15a; AtCor15b; TaWCS19; ScOMT1)*, respiratory electron transport and carbon metabolism (*AtCOX1; AtCOX2; AtAOX1a; AtSDH4; AtPDH-E1*), as well as cellular redox biochemistry (*AtGPX1; AtGPX3; AtGPX5; AtGPX6; AtAPX4; AtGR1*). *Cab* and *Lhcb* is the gene family encoding the major light harvesting proteins of PSII whereas *Lhca* represents the gene family encoding the major light harvesting proteins of PSI. *psbA* is the gene encoding the reaction centre polypeptide associated with PSII whereas *psaD* encodes the D subunit of PSI reaction centres. *PorA* and *PorB* are the genes encoding protochlorophyllide oxidoreductase A and B. *CBF3* is the gene encoding the C-repeat binding transcription factor 3 and *Cor15a* and *Cor15b* are cold-regulated genes *15a* and *15b. WCS19* is the wheat cold-regulated gene *19* whereas *OMT1* is the gene encoding O-methyltransferase1. *COX1* and *COX2* represent genes encoding subunits of mitochondrial cytochrome oxidase and *AOX1a*, the alternative oxidase1a of mitochondrial electron transport. *SDH4* is the gene encoding succinate dehydrogenase 4 of the mitochondrial electron transport and *PDH* the gene encoding the E1 subunit of the pyruvate dehydrogenase complex. Genes involved in redox biochemistry include glutathione peroxidase (*GPX*), ascorbate peroxidase (*APX*) and glutathione–disulfide reductase1 *GR*. Thus, in contrast to the report of Kim et al. [177], *CBF3* and *Cor* genes are sensitive to the redox state of the chloroplast.

However, there is considerable evidence that phytochromes also regulate the *CBF* regulon and that light quality is an important factor that governs cold acclimation and freezing tolerance [35,177,185–187]. Kim *et al.* [177] examined the integration of light and cold acclimation signaling pathways in transgenic *A. thaliana* containing four copies of a CRT/DRE element. The authors reported that phyB induced CRT/DRE reporter expression from which they concluded that light signals perceived through phytochromes regulate the *CBF* regulon and plant-freezing tolerance in a positive manner [177]. However, Franklin and Whitelam [186] reported that phytochromes repress rather than induce the expression of genes associated with the CBF regulon.

Furthermore, phytochrome regulation of the *CBF* regulon was dependent upon the ambient temperature [186]. Although exposure of *A. thaliana* to 16 °C resulted in a phytochrome-dependent upregulation of *COR* genes and an increase in freezing tolerance, this phenomenon was not observed at 22 °C [35,186]. Further mutant analysis indicated that phyB and phyD were both involved in the repression of the *CBF* regulon which counteracted the low temperature enhanced *CBF* expression as well as *COR* expression [186]. Kidokoro *et al.* [187] showed that *phyB*-regulated PIF7 (phytochrome-interacting factor 7) binds specifically to the G-box of the *DREB1C* promoter and functions as a transcriptional repressor for *DREB1C* expression when plants are not subject to abiotic stress.

Fowler *et al.* [185] reported that the low temperature-dependent expression of *AtCBF1*, *AtCBF2* and *AtCBF3* was dependent on the time during the photoperiod that *Arabidopsis thaliana* was exposed to the low temperature. The highest levels of expression were observed 4h into the photoperiod whereas the lowest were detected at the end of the photoperiod [185]. Consequently, the results of Fowler *et al.* [185] and Franklin and Whitelam [186] regarding the photoperiod-dependent expression of *CBFs* and the *CBF* regulon are consistent with the thesis that cold acclimation and plant freezing tolerance are regulated by the circadian clock from which Franklin [35] concludes that phytochromes may act as cellular temperature sensors. Furthermore, published evidence suggests that the photoreceptor- and temperature-signaling pathways involved in cold acclimation may converge with GA regulated-signaling pathways [171,172,188,189]. Indeed, the response of plants to light quality signaling is temperature- and GA-dependent [58]. Clearly, cold acclimation must be the result of a complex network of signaling pathways controlled by light quality, low temperature and plant hormones.

### 3.2. Light Intensity, Photosynthesis and Cold Acclimation

Cold-tolerant species such as winter wheat, winter rye, barley, spinach, canola and *A. thaliana* exhibit an increase in photosynthetic capacity through upregulation of carbon metabolism during cold acclimation [2,51,53,190–194]. The upregulation of carbon metabolism in cold-tolerant plant species is associated with increased expression and subsequent activities of the CO_2_-fixing enzyme Rubisco (ribulose-1,5-bisphosphate carboxylase oxygenase) [51,53,195,196], as well as enhanced activities of the cytosolic sucrose biosynthetic enzymes, cFBPase (cytosolic fructose-1,6-bisphosphatase) and SPS (sucrose phosphate synthase) [31,51,53,191,196–198] in response to low growth temperature. In addition, winter cultivars of wheat [197,199] and *A. thaliana* [193] enhance sink capacity and concomitant sucrose export to the sinks during cold acclimation [200]. Furthermore, most cereals stimulate carbon storage as fructans in the crown tissue, as well as in the leaf vacuoles during cold acclimation [201,202]. Consequently, cold acclimation of winter wheat, winter canola [191] and *A. thaliana* [193] results in enhanced *P_i_* cycling [203,204] and increased capacity for RuBP (ribulose-1,5-bisphosphate) regeneration [205] through increased utilization of phosphorylated intermediates. Furthermore, cold acclimation results in the suppression of photorespiration, thus, diverting ATP and NADPH from oxygenation to carboxylation in winter wheat [197] and also increases water use efficiency by about two-fold in winter wheat, rye and canola [51,199]. Consequently, it was suggested that cold acclimation of winter wheat and rye leads to a feed-forward upregulation of photosynthetic carbon assimilation through the global reprogramming of photosynthetic carbon metabolism [191,193,195,196]. This is supported by a detailed, comparative metabolomics study of the cold-acclimated *versus* non-acclimated *A. thaliana* [206]. In addition, the cold acclimation-induced stimulation in photosynthetic capacity is positively correlated with the development of freezing tolerance measured as LT50 [207], as well as an increased resistance to low temperature-induced photoinhibition in spinach [208,209], winter rye and winter wheat [207,210,211], *A. thaliana* [212] and canola [31].

These adjustments at the physiological, biochemical and molecular levels are associated with coordinated changes in leaf anatomy and plant phenotype [6,51,53,196,213] and induction of freezing tolerance in cold-tolerant species [8,31,198,211,214,215]. With respect to phenotypic plasticity, cold acclimation of winter wheat, winter rye, canola and spinach as well as *A. thaliana* not only results in the dwarf, compact growth habit but also altered leaf morphology and anatomy with increased leaf thickness and specific leaf weight relative to the elongated phenotype of their non-acclimated counterparts [31,51,53,196,209,216–218]. The increased leaf thickness associated with the cold-acclimated state can be accounted for by either increases in leaf mesophyll cell size [216,217] and/or increases in the number of palisade mesophyll layers [51,209]. At the ultrastructural level, cold-acclimated winter rye and *A. thaliana* exhibit an apparent increase in cytoplasmic volume and an apparent decrease in vacuolar volume [196,219]. This is accompanied by an increase in the content of sucrose and structural carbohydrates [191,196,202,204,218,220] such that the total plant biomass of the dwarf, cold-acclimated winter cultivars is equal to or greater than the nonacclimated plants which exhibit an apparently larger, elongated phenotype [6,52,53]. However, a comparable dwarf phenotype can be generated by growth of plants at warm temperatures but high light. In addition, cold acclimation of winter wheat and rye under low light conditions results in the elongated phenotype [2,6]. The historical assumption that the dwarf phenotype associated with cold acclimation reflects a growth response to low temperature is incorrect because the generation of dwarf phenotype is mimicked by growth under high light conditions. The dwarf phenotype, the enhanced photosynthetic performance and decreased susceptibility to photoinhibition of winter hardy plants previously assumed to be regulated by low temperature, is in fact, regulated by the redox state of the chloroplast measured as excitation pressure [2,6].

All photoautotrophs are predisposed to maintain a balance between energy trapped by temperature-insensitive photochemical reactions (energy source) and the energy utilized through temperature-dependent metabolism, growth and development (energy sinks) [2,4,169]. An imbalance between energy trapped through photochemistry *versus* energy either utilized through biochemistry or dissipated through nonphotochemical quenching (NPQ) will occur whenever the rate at which the energy absorbed through PSII exceeds the rates of temperature-dependent nonphotochemical processes and metabolic electron sink capacity. Such an imbalance is defined as excitation pressure which is a measure of the relative redox state of Q_A_, the first stable quinone electron acceptor of PSII reaction centres and reflects modulation of the redox state of the PQ pool and components of the intersystem photosynthetic electron transport chain (Figure 2D) [2,4,169]. The modulation of excitation pressure by acclimation to high light is mimicked by acclimation to low temperature [2,4,5]. Consequently, we have shown that excitation pressure governs the reversible conversion of the dwarf *versus* elongated phenotypes associated with cold acclimation in wheat and rye [2,4,6], high light *versus* low light phenotypes associated with cold acclimation and photoacclimation of the green algae *Chlorella vulgaris and Dunaliella salina* [2] and the cyanobacterium, *Plectonema boryanum* [221], as well as the extent of green and white sectoring in the *Arabidopsis thaliana* variegation mutant, *immutans* [222]. Since the high and low light phenotypes can be generated by chemical manipulation of the redox state of the PQ pool through inhibition of photosynthetic electron transport by either DCMU or DBMIB [176], this indicates that the phenotypic response to excitation pressure occurs independently of phytochrome. Thus, redox energy imbalances detected through modulation of excitation pressure within the chloroplast and the photosynthetic apparatus represents an extremely sensitive mechanism for all photoautotrophs to sense changes in temperature since photosynthetic redox imbalances affect nuclear gene expression through chloroplast retrograde regulation [174,178–182]. Such an “operational” redox signal is critically important for the maintenance of a functional photosynthetic apparatus in mature chloroplasts upon exposure to a changing abiotic environment [174,175].

An *Arabidopsis thaliana* genome-wide microarray analysis of gene expression in response to excitation pressure indicated that a total of 2489 transcripts, that is 10.9% of the total probes monitored on the Affymetrix *Arabidopsis* ATH1 Genome Array, were regulated by excitation pressure of which 817 were upregulated and 1672 were downregulated [5,223]. Figure 2D provides a few examples of photosynthetic and non-photosynthetic genes shown to be regulated by changes in excitation pressure in wheat (*Triticum aestivum*, *Ta*), rye (*Secale cereals*, *Sc*), *Dunaliella salina* (*Ds*), and *Arabidopsis thaliana* (*At*).

## 4. CBFs and Photosynthetic Performance

As discussed above, plant *CBFs* are integral components in the induction of the cold-acclimated state, freezing tolerance and phenotypic plasticity. The cold acclimation-induced adjustments at the physiological, morphological, biochemical and molecular levels of winter wheat and winter rye are associated with an enhanced capacity to utilize absorbed light energy and convert it to biomass and grain yield [50,51]. The apparent quantum requirements to close 50% of the PSII reaction centres and the apparent quantum requirements to induce one unit of NPQ under ambient CO_2_ conditions were about 35% and 50% higher in cold-acclimated Norstar wheat relative to its non-acclimated counterpart [50,53]. This was coupled to a 45% increase in dry biomass accumulation relative to non-acclimated counterpart [50]. Concomitantly, the cold-acclimated plants dissipate less energy as heat through NPQ because of the enhanced capacity to utilize and store absorbed energy as biomass. This means that in the cold-acclimated state, the winter cereals are able to trap a greater percentage of the incident radiation through photochemistry and convert this energy into biomass and seed production. Comparable results for cold acclimation-induced increases in biomass and concomitant increases in the apparent quantum requirements for PSII closure and NPQ induction under ambient CO_2_ conditions were reported for Musketeer winter rye [51,53].

What regulates the increased energy conversion efficiency observed for cold-acclimated winter cereals? Compared to non-acclimated wild-type (WT), the *BnCBF17*-overexpresser (*BnCBF17*-OE) grown at 20 °C mimicked cold-acclimated WT canola with respect to increased biomass and specific leaf weight (SLW), compact dwarf phenotype, increased light saturated rates of photosynthesis and photosynthetic electron transport, improved water use efficiency (WUE) and enhanced levels of key Calvin Cycle enzymes and components of photosynthetic electron transport, at ambient CO_2_[51,53]. *BnCBF17*-overexpression as well as cold acclimation of WT canola increased the dry biomass by 25%–35% relative to non-acclimated WT at ambient CO_2_ which were associated with increased amount of key regulatory photosynthetic enzymes such as stromal-localized Rubisco (rbcL) and cFBPase important in regulating sucrose biosynthesis [51]. These results for the *BnCBF17*-OE and cold-acclimated canola are comparable to the enhanced photosynthetic performance reported for cold-acclimated winter wheat and winter rye [53] and consistent with the report of Savitch *et al.* [31] who reported that the *BnCBF17*-OE exhibited significant enhancement in the gene expression as well as enzyme activities of Rubisco, SPS and cFBPase. Concomitantly, this was associated with an increased quantum requirement to close PSII reaction centres and to induce energy dissipation by NPQ in cold-acclimated and the *BnCBF17*-OE relative to WT controls. This indicates that compared to non-acclimated WT, *BnCBF17*-OE as well as cold-acclimated WT canola exhibit a comparable enhanced capacity to utilize absorbed light energy and convert it to biomass with a concomitant decreased reliance on NPQ to dissipate absorbed energy for photoprotection. Tobacco plants overexpressing *A. thaliana CBF* exhibited increased freezing tolerance and had a higher photochemical efficiency of PSII during cold acclimation period relative to WT plants [224]. Genetic alterations which influence chloroplast development, such as those regulated by *MutS HOMOLOG1* (*MSH1*), induced programmed changes in phenotypic plasticity of six different plant species [225]. Recently, we have shown by genome-wide transcriptome analyses of *A. thaliana* that *CBF3* transcript levels are regulated by excitation pressure (Figure 2D) [223]. We conclude that CBFs are critically important factors, not only in the regulation of plant freezing tolerance and phenotypic plasticity, but also in the governance of photosynthetic performance and energy conversion efficiency. Therefore, we suggest that CBFs may represent master regulators which integrate phenotypic plasticity, cold acclimation and hormonal homeostasis with photosynthetic performance and energy conversion efficiency which results in enhanced biomass production and seed grain yield [51] under suboptimal growth conditions.

## 5. CBFs as Master Regulators

Historically, research focused on cold acclimation and freezing tolerance has focused on changes in the physical structure of the cell membrane as a primary sensor involved in acclimation to low temperature [8,165,216,226]. Low temperature-induced increase in cell membrane viscosity has been shown to activate two-component histidine kinases which stimulate the expression of specific fatty acid desaturases involved in the modulation of membrane fluidity in cyanobacteria [227,228]. Modification of the physical properties of the cell membranes also activates Ca^2+^ channels which results in the accumulation of Ca^2+^ in the cytosol. Such a low temperature-induced Ca^2+^ signal occurs rapidly upon exposure to low temperature stress [229–231]. This accumulation of Ca^2+^ in the cytosol activates a protein kinase which phosphorylates ICE1 [8] which is necessary to induce the expression of *CBFs* and initiate the expression of *COR* genes necessary to acquire freezing tolerance [8,14,15–17,35,186,232,233]. A unique feature of all plants and photosynthetic micro-organisms is that they are photoautotrophic. Thus, these organisms are dependent upon light both as a signal to regulate plant development and reproduction through photoreceptors such as the phytochromes and cryptochromes [234], as well as an energy source that regulates primary C, N and S assimilation [5,235,236]. The regulation of the phytochrome-dependent, photomorphogenic signal transduction pathways [35,186], as well as the retrograde sensing/signaling pathways between the chloroplast and the nucleus involved in remodeling of the photosynthetic apparatus in response to changes in excitation pressure, are both signaling networks which govern plant plasticity in response to an ever-changing environment. Consequently, we suggest that plants do not exhibit a single low temperature sensor, but rather, they integrate information regarding changes in temperature through changes in the redox state of the photosynthetic electron transport chain, phytochrome, as well as specific cell membrane, low temperature sensors in order to establish the cold-acclimated state.

**Figure 3:**
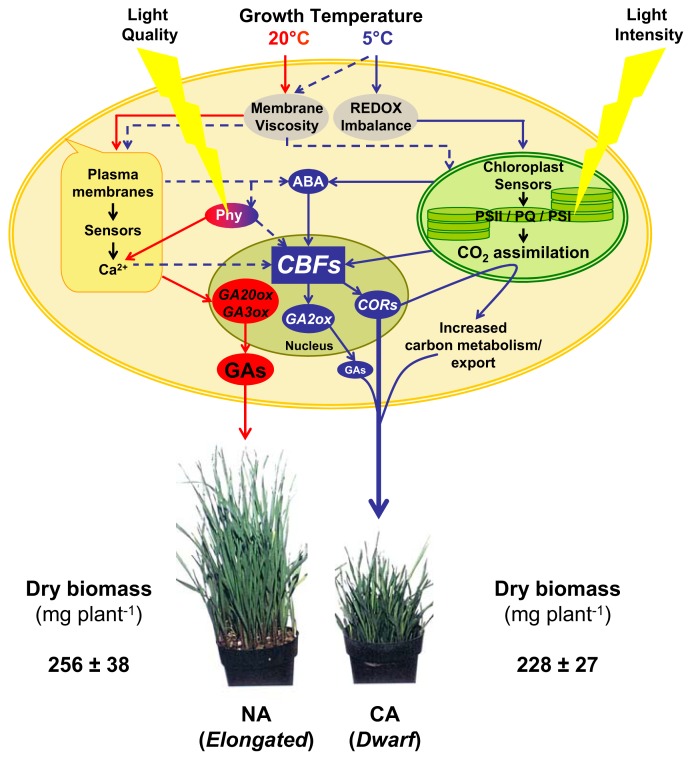
CBFs as integrators of cold acclimation: a schematic model of plant responses to growth temperature of 20 °C and 5 °C (see text for details). ABA—abscisic acid; CA—cold-acclimated; *CBFs*—*C-repeat binding factors; COR*—*cold regulated*; Gas—growth-active gibberellins; *GA2ox*—*GA2 oxidase; GA3ox*—*GA3 oxidase; GA20ox*—*GA20 oxidase*; NA—non-acclimated; Phy—phytochromes; PQ—plastoquinone; PSI or PSII—photosystem I or II.

There appears to be a consensus in the literature that ICE1 stimulates *AtCBF3* transcription which, in turn, activates the *COR* family required for cold acclimation. Concomitantly, this cold acclimation process results in the development of a dwarf phenotype [14,16–18]. Overexpression of *CBFs* mimics the cold acclimation process, as well as the phenotypic plasticity and enhanced photosynthetic performance associated with cold acclimation [2,6,14,16–18,31,51,53]. We reported that *COR* gene expression, the development of the dwarf plant phenotype, in addition to photosynthetic performance are regulated by the chloroplast redox status measured as excitation pressure [2,6]. Furthermore, Figure 2D illustrates that the expression of *AtCBF3*, *AtCor15a*, *AtCor15b* appeared to be governed by chloroplast excitation pressure modulated either by low temperature or high light. Thus, in Figure 3, we present a simplified schematic model which attempts to illustrate a central role for *CBFs* in integrating input signals generated by changes in plasma membrane viscosity due to low temperature, as well as changes in light quality sensed through the photoreceptor, phytochrome, but also input signals due to changes in chloroplast redox imbalance, generated either by modulation of growth temperature and/or growth irradiance.

Growth of winter rye plants at 20 °C (“red” pathway) is likely regulated by photoreceptors such as phytochrome as well as the plasma membrane and translated in the downstream mode with Ca^2+^ as second messenger which ultimately leads to the appearance of a typical elongated phenotype. This is dependent upon the upregulation of *GA20ox* and *GA3ox* genes which result in high levels of growth-active GAs. In contrast, growth of winter rye plants at a cold acclimation temperature of 5 °C (the “blue” pathway) to a comparable developmental stage as 20 °C-grown plants is sensed by changes in plasma membrane viscosity (dashed blue line pathway), phytochrome and chloroplast redox imbalance (solid blue line pathway). The increase in plasma membrane viscosity increases Ca^2+^ levels which eventually leads to upregulation of ABA biosynthesis. ABA biosynthesis may also be induced by a redox imbalance in chloroplast which causes changes in the redox state of the photosynthetic electron transport chain (PSII/PQ/PSI). The increase in ABA biosynthesis activates *CBF* gene expression which can also be activated independently of ABA, likely directly by chloroplast redox imbalance. Upregulation of *CBF* genes causes an increase in the expression of *COR* and *GA2ox* genes. Upregulation of *GA2ox* genes reduces the amount of growth-active GA genes which is integral in the generation of the dwarf phenotype. Upregulation of *COR* genes not only increases cold and freezing tolerance, but also enhances photosynthetic performance via its effect on increased CO_2_ assimilation, photosynthetic carbon metabolism and carbon export. This results in plants which exhibit a dwarf phenotype at 5 °C and a dry biomass comparable to plants grown at 20 °C. The comparable dry mass between the elongated and dwarf phenotype is accounted for by increased leaf thickness, decreased cellular water content and increased size of crown tissue in cold-acclimated *versus* non-acclimated plants [2]. Although further research is necessary, we believe that the proposed model provides a framework through which the model can be tested experimentally.

## 6. Conclusions

In summary, we suggest that CBFs are master regulators of cold acclimation which integrate both the upstream and downstream signals. We propose that input sensors including chloroplast redox imbalance, phytochromes, as well as membrane viscosity are integrated by CBFs, likely directly via upregulation of either ABA metabolism, or indirectly through chloroplast redox imbalance. Furthermore, we propose that upregulation of *CBF* genes regulates the phenotype and physiological properties of cold-acclimated plants via the two output signals: upregulation of *COR* genes to induce freezing tolerance and photosynthetic and respiratory genes involved in carbon metabolism, in addition to the upregulation of *GA2ox* genes to remove growth-active GAs. This results in a dwarf phenotype that exhibits enhanced photosynthetic performance and high biomass coupled with enhanced seed yield [51]. We cannot discount the possible roles of other plant hormones in increased freezing tolerance following a period of cold acclimation. SIZ1-induced inhibition of SA accumulation may be necessary for cold acclimation process [164], and both CKs and BRs may be involved in CBF-regulated cold acclimation. However, a consensus regarding the regulation of *CBFs* by SA, CKs and BRs remains to be established. We suggest that, due to their photoautotrophic nature, plants probably do not exhibit a single low temperature sensor, but rather, through an integrated network, information regarding changes in temperature, light intensity and light quality are sensed through chloroplast redox imbalance, photoreceptors such as phytochrome, as well as specific cell membrane, low temperature sensors in order to establish the cold-acclimated state. This is consistent with the thesis that the chloroplast has a dual function: not only is it the primary energy transformer for all photoautotrophs, it also acts as a major sensor for detecting changes in the environment [2].
